# Cohort Profile: The Africa Medical and Behavioral Sciences Organization (AMBSO) Population Health Surveillance (APHS) in rural, semi-urban and urban Uganda

**DOI:** 10.1093/ije/dyac164

**Published:** 2022-08-13

**Authors:** Stephen Mugamba, Leo Ziegel, Robert M Bulamba, Emmanuel Kyasanku, Katarina Johansson Århem, Carl Fredrik Sjöland, Amanda P Miller, Gertrude Nakigozi, Grace Kigozi Nalwoga, Stephen Watya, Noah Kiwanuka, Joseph Kagaayi, Deusdedit Kiwanuka, William Ddaaki, Jennifer A Wagman, Godfrey Kigozi, Anna Mia Ekström, Fred Nalugoda

**Affiliations:** Africa Medical and Behavioral Sciences Organization, Wakiso, Uganda; Makerere University Walter Reed Project, Kampala, Uganda; Department of Global Public Health, Karolinska Institutet, Stockholm, Sweden; Africa Medical and Behavioral Sciences Organization, Wakiso, Uganda; Africa Medical and Behavioral Sciences Organization, Wakiso, Uganda; Department of Global Public Health, Karolinska Institutet, Stockholm, Sweden; Department of Infectious Diseases, Karolinska University Hospital, Stockholm, Sweden; Department of Global Public Health, Karolinska Institutet, Stockholm, Sweden; Department of Epidemiology, Fielding School of Public Health, University of California Los Angeles, Los Angeles, CA, USA; Africa Medical and Behavioral Sciences Organization, Wakiso, Uganda; Africa Medical and Behavioral Sciences Organization, Wakiso, Uganda; Africa Medical and Behavioral Sciences Organization, Wakiso, Uganda; Africa Medical and Behavioral Sciences Organization, Wakiso, Uganda; School of Public Health, Makerere University, Kampala, Uganda; Africa Medical and Behavioral Sciences Organization, Wakiso, Uganda; School of Public Health, Makerere University, Kampala, Uganda; Africa Medical and Behavioral Sciences Organization, Wakiso, Uganda; Africa Medical and Behavioral Sciences Organization, Wakiso, Uganda; Department of Community Health Sciences, Fielding School of Public Health, University of California Los Angeles, Los Angeles, CA, USA; Africa Medical and Behavioral Sciences Organization, Wakiso, Uganda; Department of Global Public Health, Karolinska Institutet, Stockholm, Sweden; Department of Infectious Diseases, Södersjukhuset, Stockholm, Sweden; Africa Medical and Behavioral Sciences Organization, Wakiso, Uganda

Key Features

- The Africa Medical and Behavioral Sciences Organization (AMBSO) Population Health Surveillance (APHS) is an open, longitudinal, population-based cohort including persons aged 13–80 years in diverse settings of rural, semi-urban and urban communities in Hoima and Wakiso districts in the Western and Central regions of Uganda.
- APHS is the first health and demographic surveillance system (HDSS) in Uganda that includes an urban cohort.
- Data are collected through face-to-face questionnaires covering a broad range of health-related issues including communicable and non-communicable diseases, risk factors and health determinants. Physical measurements and biological samples, e.g. for HIV status, are also collected.
- A baseline census was conducted in 2018/19 including 20 210 individuals. Of all 10 929 individuals eligible for the baseline survey, 4606 participated (42.1% participation rate). Follow-up rounds are conducted annually with possible mid-round phone surveys, such as one performed during the COVID-19 pandemic.
- Interested researchers may request access to data for analysis if appropriate funding is made available and the Ugandan partners are involved. The point of contact for data requests and collaborations is the APHS Principal Investigator via [info@ambso.org].

## Why was the cohort set up?

The Africa Medical and Behavioral Sciences Organization (AMBSO) Population Health Surveillance (APHS) was established as an open, longitudinal, population-based cohort in 2018. APHS is the first health and demographic surveillance system (HDSS) in Uganda to include urban sites in addition to semi-urban, rural and fishing communities, and tracks health outcomes and their determinants in diverse communities.

APHS focuses on under-researched and emerging public health issues in Uganda, including mental health, substance use, gender-based violence (GBV), violence against children, food insecurity, health behaviours, disability and emerging diseases. Data collection from census activities and surveys target community types which exhibit considerable variation in risk behaviours and health conditions. As such, the cohort reflects the diversity of the Ugandan population and allows for stratification by district and community type. AMBSO complements this research with qualitative work aiming to keep the APHS dynamic and responsive to changing needs. Uganda represents one of the most rapidly urbanizing countries in the world and faces a growing burden of both communicable and non-communicable diseases.^1^^,^^2^

AMBSO is a Ugandan non-profit research and service organization that maintains APHS with the objectives of: (i) monitoring trends in communicable and non-communicable diseases, reproductive health, and determinants including demographic, social, behavioural and economic factors; (ii) providing a platform for community trust building, health service delivery and patient referral; (iii) capacity building for researchers, students and fellows; (iv) maintaining an archive of interview data and biological samples; and (v) providing a platform for future research studies.

## Who is in the cohort?

### APHS locations

**Figure 1:**
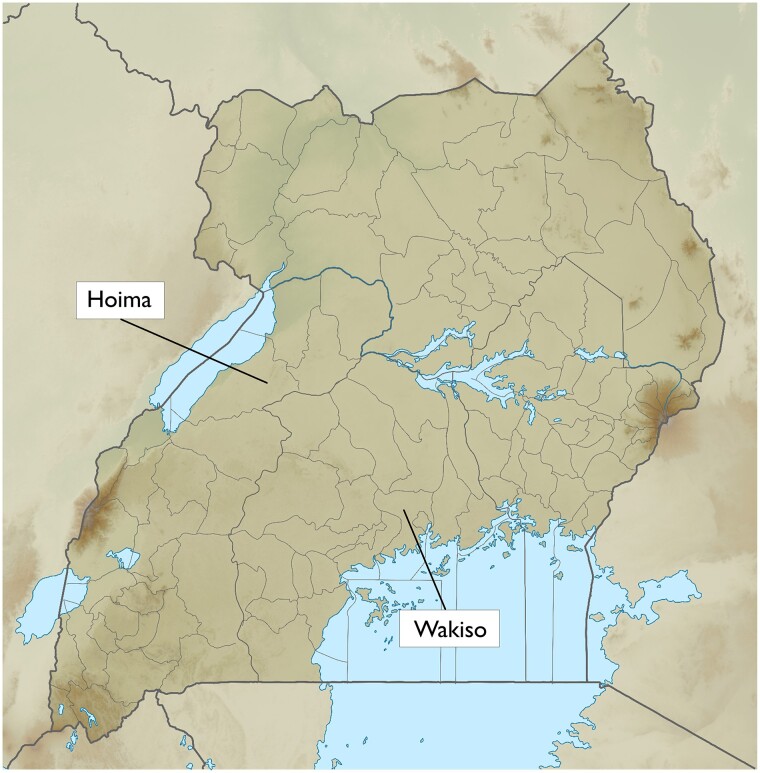
Map of Uganda with Wakiso and Hoima districts, labelled. Adapted using material from Wikimedia Common [https://commons.wikimedia.org/wiki/File:Uganda_relief_map.svg] by C1MM, NordNordWest; and [https://commons.wikimedia.org/wiki/File:Hoima_District_in_Uganda.svg] by OpenStreetMap contributors, Jarry1250, NordNordWest—under licence by CC-BY-SA-3.0-DE [https://creativecommons.org/licenses/by-sa/3.0/de/legalcode] and CC-BY-SA 3.0 Unported [https://creativecommons.org/licenses/by-sa/3.0/deed.en]

**Figure 2:**
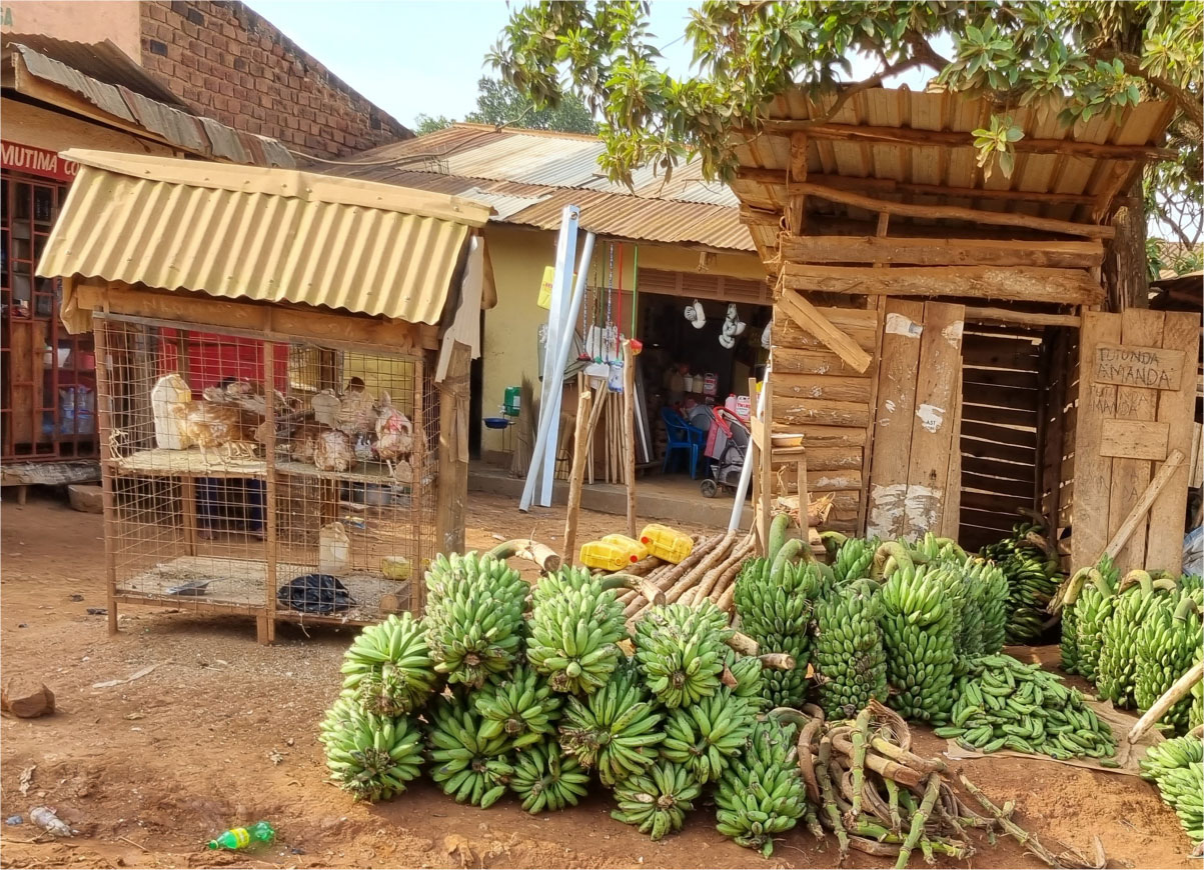
Matooke (East African Highland banana) is a staple food in Central and Western Uganda, with chicken being a common source of animal protein throughout the country. The picture shows a typical retail shop

**Figure 3:**
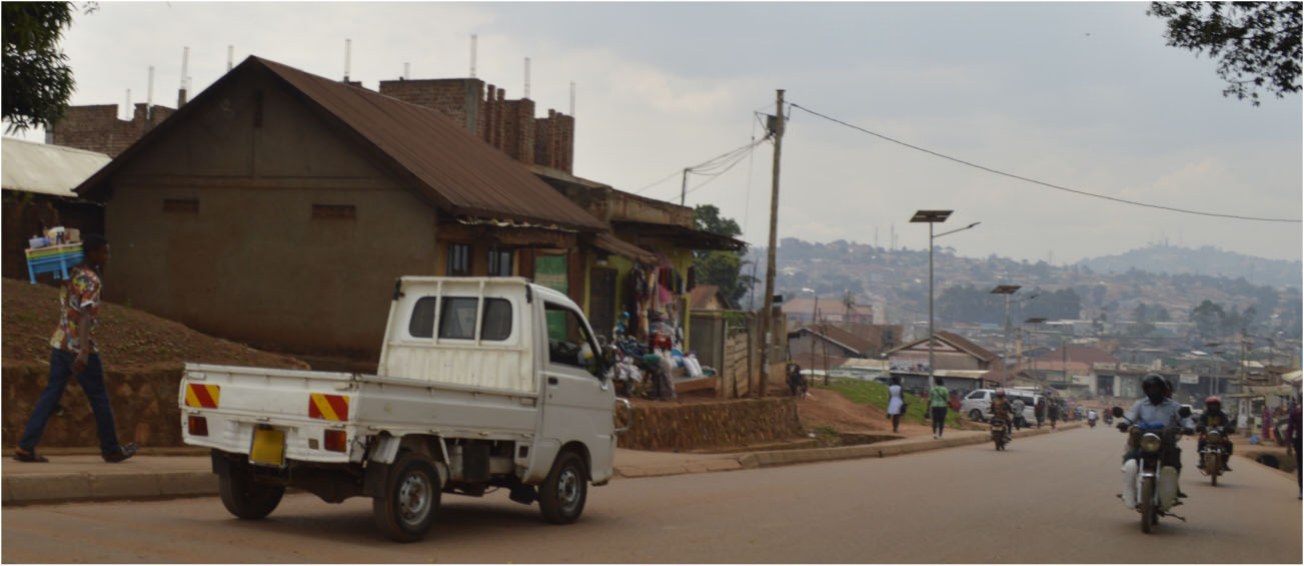
Urban road scene in Wakiso

APHS operates in two Ugandan districts: Wakiso, which surrounds the capital city of Kampala, located in Uganda’s Central region; and Hoima in the Western region, 200 km northwest of Kampala (see Figure 1). In both regions, urban, semi-urban and rural communities are surveyed. At the initial census, areas were selected through a community mapping exercise comparing the population structure of areas with different distances from urban centres and varying degrees of cultivation. The aim was to obtain a representative sample of households typical for the different community types (rural, semi-urban, urban) and with varying socioeconomic status (see Figures 2 and 3 for representative road scenes).

Selected areas were defined to include all households within a set distance from a major road passing through the community. The geographical area of each community differs depending on community type and population density, in order to cover a similar number of households. In each area, household buildings were mapped using GPS coordinates. Similarly, major and minor roads, health facilities, places of worship, general stores, recreation sites and other relevant structures were mapped in a wider area approximating an average walking distance from the community.

The Wakiso cluster consists of three communities: Kazo (urban), Lukwanga (semi-urban) and Sentema (rural). Wakiso district has the highest annual population growth rate of all Ugandan districts (6.2% vs 3.3% national average) due to rapid urbanization and high fertility.^3^ The population has a Christian majority (Catholic and Protestant denominations) with a substantial Muslim minority. The main spoken local languages are Luganda and English (see Table 1).

**Table 1:** Full demographics of 2018/19 baseline survey of the Africa Medical and Behavioral Sciences Organization (AMBSO) Population Health Surveillance (APHS)

	Rural cohort *n* = 1502		Semi-urban cohort *n *= 1330				Urban cohort *n* = 1774		Total APHS cohort *n* = 4606		
Mean number of individuals per household	4.2		3.7				3.4		3.7		
Sex											
− Male	674	45%	618	46%			723	41%	2015	44%	
− Female	828	55%	712	54%			1051	59%	2591	56%	
Male: female ratio	81:100		87:100				69:100		78:100		
Age in years											
−13–17	243	16%	186	14%			257	14%	686		15%
−18–19	120	8%	101	8%			154	9%	375		8%
−20–29	403	27%	456	34%			744	42%	1603		35%
−30–39	252	17%	270	20%			354	20%	876		19%
−40–49	224	15%	163	12%			160	9%	547		12%
−50–59	144	10%	94	7%			67	4%	305		7%
−60+	116	8%	60	5%			38	2%	214		5%
Marital status											
−Single	766	51%	639		48%		971	55%	2376		52%
−Married	736	49%	691		52%		803	45%	2230		48%
Proportion women of reproductive age (15–49 years)	610/828	74%	595/712		84%		928/1051	88%	2133/2591		82%
Women currently pregnant	56/828	7%	60/712		8%		65/1051	6%	181/2591		7%
Religion											
−None	3	0.2%	13			1%	7	0.4%	23		0.5%
−Catholics	659	44%	545			41%	606	34%	1810		39%
−Protestants	523	35%	401			30%	495	28%	1419		31%
−Saved/Pentecostals	125	8%	136			10%	260	15%	521		11%
−Muslims	180	12%	221			17%	379	21%	780		17%
−Seventh Day Adventists	6	0.4%	10			1%	18	1%	34		1%
−Other (traditional etc)	6	0.4%	4			0.3%	9	0.5%	19		0.4%
Education											
−None	72	4%	45		3%		33	2%	150		3%
−Primary	836	56%	674		51%		675	38%	2185		47%
−Secondary and above	594	40%	611		46%		1066	60%	2271		50%

The Hoima cluster also consists of three communities: Isaka-Kijjungu (urban), Butema (semi-urban) and Kitoba (rural). A fishing community (Tonya) on the shore of Lake Albert has been mapped and qualitatively surveyed, and is planned for future inclusion in APHS. The urban community in Hoima city profits from oil industry developments near Lake Albert. Dominant denominations in the area are Catholicism and Protestantism. The main languages spoken are Runyoro, Kiswahili and English.

### APHS study participants

**Figure 4:**
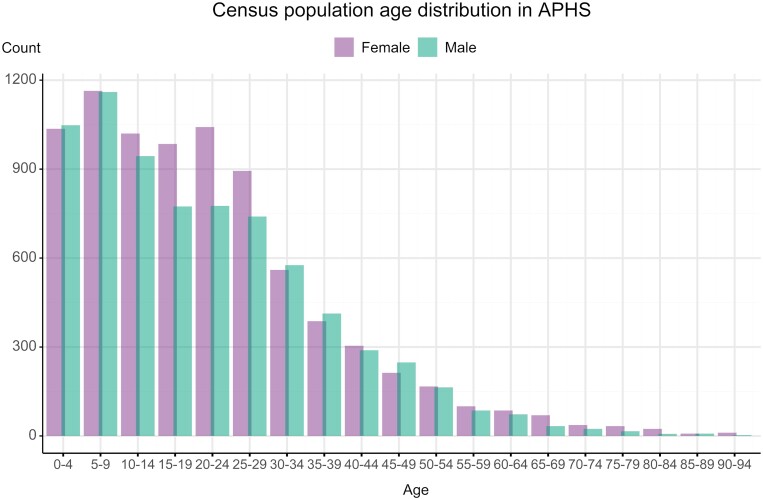
Population age distribution of 2019 baseline census of the Africa Medical and Behavioral Sciences Organization (AMBSO) Population Health Surveillance (APHS)

The first round of the APHS was conducted between May 2018 and July 2019. During this period, 20 210 individuals (48.3% males, 51.8% females) from 5524 households were censused within the three community types: 5156 individuals in rural, 5483 in semi-urban and 9571 in urban communities (see Figure 4 for age distribution). Of the census participants, 10 929 (54.1%) were 13–80 years old and thus eligible for survey participation. In total, 4606 individuals (43.8% males, 56.2% females) successfully participated in the survey, giving a 42.1% participation rate (see Table 1). Mean age was 29.2 years among all eligible censused individuals, and 30.4 years among those who actually participated.

## How often have they been followed up?

### Survey plan

Survey rounds with census and sample collection are conducted annually. Baseline and follow-up surveys are conducted in parallel 1–2 months after census. Through community engagement (see below), eligible individuals aged 13–80 years are invited to the data collection hub. Individuals participating for the first time are administered a detailed baseline questionnaire, and follow-up participants receive a shorter follow-up questionnaire, with a 50–60% target inclusion rate of censused individuals for either survey. During the COVID-19 pandemic, a limited census mapping and mid-round telephone survey were performed (described below). See Table 2 for a summary of data collection procedures.

**Table 2:** Data collection procedures with community engagement in mind—the Africa Medical and Behavioral Sciences Organization (AMBSO) Population Health Surveillance (APHS) model

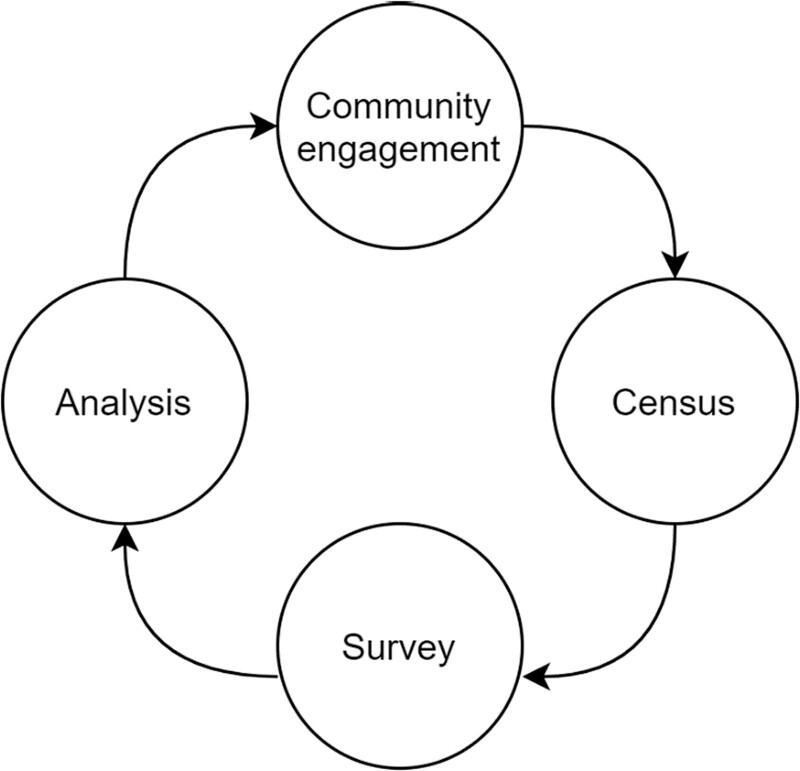	**Community engagement**: meet local leaders, hold community meetings, update collaborating partners **Census**: collect geographical data, obtain informed consent, administer census questionnaire, update paper maps on in- and out-migration/new vs abandoned households, record household births and deaths S**urvey**: obtain informed consent, administer the survey questionnaire, conduct physical measurements, take and analyse blood and urine samples, provide HIV testing and counselling, screen and treat sexually transmitted infections **Data analysis**: prepare results for community feedback, scientific publication and wider dissemination to national and international stakeholders

### Census

Each census captures all currently inhabited households in the pre-defined community and collects information on residence status (visitor, transient, permanent), age, sex, relationship of individuals to the household head, dwelling characteristics, relevant possessions, water sources and more. Data are preferably captured from the head of household, but if no household member is present during one of several visits, basic characteristics are obtained from neighbours or community leaders. A new census is performed before each survey round.

### Baseline survey

Baseline surveys include full questionnaires covering all modules detailed below (Table 3), with a focus on lifetime exposure and non-transient characteristics.

**Table 3:** Data collected by Africa Medical and Behavioral Sciences Organization (AMBSO) Population Health Surveillance (APHS)

Module	Topics
Census questionnaire	Number of household members, orphanhood, disability, births, deaths, dwelling characteristics, socioeconomic status, mobility, water, sanitation
Survey questionnaire	Access to health services and health-seeking behaviours Nutrition, food security Non-communicable diseases: diabetes, cardiovascular disease, cancer, depression, anxiety, smoking, alcohol and substance use Symptoms or diagnosis of infectious diseases: HIV, syphilis, tuberculosis, hepatitis B and COVID-19 (since 2020) Childhood and adult immunization status. Knowledge, attitudes and beliefs surrounding infectious diseases and immunization Reproductive health and sexual behaviours: sexual practices, sexual risk behaviours, pregnancy and outcomes, family planning practices, access to reproductive and sexual health care services, gender-based violence (GBV), violence against children. Human papilloma virus (HPV) immunization and in-depth prostate cancer questions
Measurements collected during survey	Blood pressure, height, weight, body mass index (BMI), waist-to-hip ratio
Samples collected during survey	Blood: rapid HIV test, rapid syphilis test, random blood glucose test, two separate aliquots for archives Urine for human chorionic gonadotropin (hCG) test
Qualitative data (collected 2018/19)	HIV testing, disclosure of HIV status, health care use after HIV test; knowledge, attitudes and beliefs about HIV pre-exposure prophylaxis (PrEP) Voluntary medical male circumcision, family planning practices, sex work in the community Immunization of children, health and health services in the community, non-communicable diseases, lifestyle, eating habits, alcohol and drug use, mental health and GBV

### Follow-up survey

Follow-up surveys are administered to participants who have already taken part in the baseline survey. The questionnaire contains a subset of baseline survey modules, focusing on the past 12-month exposure (i.e. since the previous round of data collection). Follow-up aims at an 85% retention rate between rounds in rural, 75% in semi-urban and 65% in urban communities. The first full follow-up round began in November 2019, was interrupted during the COVID-19 pandemic, then subsequently resumed and completed in March 2021.

### Phone survey

The APHS protocol allows for mid-round phone surveys to obtain time-sensitive information in case of unforeseen events. The first phone survey was conducted during the COVID-19 pandemic between June and August 2020 in the Wakiso cluster. Ordinary data collection was disrupted for the cluster, and individuals were re-surveyed with an adapted survey including a COVID-19 module, aiming to capture immediate effects of the pandemic and the nationwide lockdown on health outcomes, socioeconomics and access to health services.

### Community and stakeholder involvement

At the commencement of each round, residents in the APHS communities are provided with health education messages at stakeholder engagement meetings, town halls and village meetings, covering topics such as general community health, reproductive health, non-communicable diseases, infectious diseases, alcohol and drug use and GBV prevention. Study findings from the APHS are disseminated through similar meetings as well as sensitization meetings for community leaders and the general population. The community engagement activities are supported by a network of trained community health workers, local leaders and the Community Advisory Board (CAB), an independent committee that represents the communities’ interests.

### Administrative support

The APHS cohort is supported by a multilingual team of accredited epidemiologists, biostatisticians, counselling psychologists, laboratory technologists, medical doctors, nurses and social workers employed by AMBSO. All staff are given continuous training on Good Clinical Practice and Human Subjects Protection. Regular medical education sessions are also held with staff to ensure continuous capacity building, offer specialized competence strengthening and also discuss any specific clinical or research-related incidents and processes.

Field data collection is facilitated through appropriate transportation of items and supplies to and from the field sites in AMBSO vehicles. The field team is equipped with mobile laboratory supplies, sample storage equipment, field laptops and stationery.

## What has been measured?

### Questionnaire data collection

Following a census, all eligible study participants are invited to nearby community data collection hubs which are open for 4–6 weeks for interviews and biological samples collection. At these community hubs, additional eligibility screening is performed alongside obtaining informed consent. Consenting participants are assigned anonymized IDs (identifiers), and the baseline or follow-up interviews are conducted face to face with confidentiality towards other participants. Same-gender research assistants administer the questionnaires, which include questions on demographics, health-seeking behaviour, sexual and reproductive health and rights (SRHR), mental health, communicable and non-communicable diseases, exposure and perpetration of GBV, alcohol and drug use, and other relevant topics (see Table 2). Data are collected on AMBSO laptops using Microsoft Access or on paper for digitization by the on-site primary data management team. All collected data undergo real-time editing and review for quality control to identify inconsistencies while participants remain available at the hub. Real-time backup is performed each day to portable disks, which are transported to the AMBSO data department for backup and storage on secure servers. Following this, the data undergo additional review before being saved to a final dataset. Data cleaning continues throughout the analysis process.

Participants are encouraged to identify themselves using a national ID card instead of only their names to avoid impersonation, to maintain confidentiality and personal integrity and to ensure correct delivery of test results. Fingerprints and photographs of participants are kept, with their permission, to ensure correct registration at follow-up. Participants are compensated for their time and transportation costs.

### Anthropometrics and biological sample collection

Measurements collected from consenting individuals include weight, height, waist-hip circumference and blood pressure. Participants are also invited to provide a 10-ml blood sample, collected by trained phlebotomists. All individuals who provide samples for HIV testing are offered pre- and post-test counselling, and those who test positive for HIV and are not yet on antiretroviral treatment are referred to HIV care. Participants who test positive for syphilis are offered free treatment by the study team. Screening and management of HIV and syphilis are done according to the guidelines of the Uganda Ministry of Health.

After each day in the field, the samples are placed in ice-packed cool boxes for transport to AMBSO’s main laboratory for further processing, with centrifuging and separation into aliquots for storage and testing. Samples are archived for future research.

### Data and sample storage

All participant data are pseudonymized and linked only to de-identified participant and household IDs. Thereafter, the data are kept in restricted rooms or password-protected computers accessible only by designated AMBSO data managers/editors. Biological samples are stored in -80 °C freezers at AMBSO’s main laboratory in Kampala. Duplicate aliquots for future use are stored at Mulago University School of Biomedical Sciences Laboratory and the Ministry of Health Central Public Health Laboratories.

## What has been found?

Findings from APHS have already resulted in several scientific publications and abstracts that have been presented at national and international conferences.

In the baseline survey round, nearly one in four (24%) surveyed households reported food insecurity; among men, food insecurity was associated with self-reported perpetration of intimate partner violence.^4^ In this initial round, food insecurity was assessed using a single question; in subsequent rounds, it has been measured using a validated battery of questions from the Food Insecurity Experience Scale (FIES).^5^

Qualitative research on how mental health is conceptualized in the population found that environmental and societal stressors were identified as primary underlying causes of poor mental health, and supernatural explanatory models frequently encountered in previous research in Uganda were notably absent.^6^ To ensure proper screening for depression in participants’ native language, the Patient Health Questionnaire-9 (PHQ-9) was translated into Luganda (spoken in Wakiso) and Runyoro (spoken in Hoima) and psychometrically assessed, demonstrating satisfactory construct validity and internal consistency.^7^ In the aforementioned COVID-19 telephone survey, we found that women’s alcohol use had gone down but their recent exposure to GBV had increased during the lockdown.^8^

There are also several ongoing projects related to mental health, health-seeking behaviours, food insecurity, COVID-19, HIV (see Table 4 for baseline statistics), sexual risk behaviours, GBV and violence against children, which will result in forthcoming publications.

**Table 4:** HIV and syphilis statistics from 2018/19 baseline survey of the Africa Medical and Behavioral Sciences Organization (AMBSO) Population Health Surveillance (APHS)

Indicator	Prevalence	
HIV	Total	7.3%
	Male	5.0%
	Female	9.0%
– Urban	Male	4.3%
	Female	8.2%
– Rural	Male	5.5%
	Female	7.9%
– Semi-urban	Male	5.5%
	Female	12%
Syphilis	Total	7.0%

## What are the main strengths and weaknesses?

APHS is unique as it includes both rural and urban communities in Uganda. This allows for comparison of behaviours and health outcomes across settings and capture of trends over time in a rapidly urbanizing environment.^9–11^ The wide age range of participants enables the study of health outcomes across the lifespan, from adolescence to old age. Internationally validated measurements are used to ensure comparability of APHS results to results from other HDSS, both nationally and internationally. The scope of health issues currently covered in the APHS fills gaps in the existing evidence base regarding non-communicable diseases in sub-Saharan Africa.^12^

APHS was conceived of and is managed by a team of experienced Ugandan researchers who established the cohort with locally mobilized seed money. The APHS leadership has considerable experience from other large research projects in Uganda, and a majority are affiliated or hold co-appointments with academic institutions in the country. The strong local foundation facilitates acceptability and trust towards the AMBSO research team among study communities.

The versatile and comprehensive nature of APHS and the AMBSO infrastructure can facilitate collaboration across disciplines and research institutes, allowing generations of rich analyses and providing a strong infrastructure for the integration of interventions and scale-up of service delivery. The study cohort is adaptable to changing and emerging priorities, which is evidenced by the quick approval and implementation of a COVID-19 phone survey during the pandemic lockdown in 2020.

Challenges include the open nature of the cohort and high rates of in- and out-migration in some of the communities, which complicate retention between rounds. To maintain a study of this size requires substantial human and monetary resources, a constant challenge for all HDSS sites in low- and middle-income settings.

## Can I get hold of the data? Where can I find out more?

There is room for new collaborators and partners with interest in exploring health outcomes in Uganda in line with AMBSO’s goals.^13^ Interested researchers may request access to data for analysis by completing a data request form given that appropriate funding is made available and that the Ugandan researchers are involved. Requests are reviewed and approved by the AMBSO scientific committee. The point of contact for data requests and collaborations is the APHS Principal Investigator Dr Stephen Watya at [info@ambso.org].

## Ethics approval

The APHS protocol was reviewed and approved by Clark International University Research and Ethics Committee and registered with the Uganda National Council for Science and Technology. Renewed approvals are sought annually. All participants undergo an informed consent process which includes oral and/or written information. Consent is recorded as a signed document or an audio file, and is retained for those who choose to participate. For illiterate participants, a witness is required for consent, of which additional records are retained. Children below 18 years of age, who assent to participate, require consent from a legal guardian. Emancipated minors are treated in the same manner as adults. Any individual identified during the data collection process to be in need of health care or counselling is referred to appropriate government health care services by the study team.

## Data Availability

See ‘Can I get hold of the data?’ above.
